# Oriental Medicine Samhwangsasim-tang Alleviates Experimental Autoimmune Encephalomyelitis by Suppressing Th1 Cell Responses and Upregulating Treg Cell Responses

**DOI:** 10.3389/fphar.2017.00192

**Published:** 2017-04-18

**Authors:** Min J. Lee, Jong H. Choi, Sung J. Lee, Ik-Hyun Cho

**Affiliations:** ^1^Department of Science in Korean Medicine and Brain Korea 21 Plus Program, Graduate School, Kyung Hee UniversitySeoul, South Korea; ^2^Department of Convergence Medical Science, College of Korean Medicine, Kyung Hee UniversitySeoul, South Korea; ^3^Department of Neuroscience and Physiology, Dental Research Institute, School of Dentistry, Seoul National UniversitySeoul, South Korea; ^4^Institute of Korean Medicine, College of Korean Medicine, Kyung Hee UniversitySeoul, South Korea

**Keywords:** Samhwangsasim-tang, experimental autoimmune encephalomyelitis, Th1 cells, Treg cells, anti-inflammation

## Abstract

Oriental medicine Samhwangsasim-tang (SHSST) has traditionally been used in East Asia to treat hypertension and its complications. However, little is known about its potential value regarding the treatment of chronic inflammatory diseases such as multiple sclerosis (MS). In this study, we investigated whether SHSST has a beneficial effect in treating myelin oligodendrocyte glycoprotein-induced experimental autoimmune encephalomyelitis (EAE). Onset-treatment with SHSST was found to alleviate neurological symptoms as well as demyelination and glial activation in the spinal cords from the EAE mice. The SHSST also attenuated the mRNA or protein expression of pro-inflammatory cytokines (interleukin-1beta and tumor necrotic factor-alpha); chemokines (RANTES, monocyte chemotactic protein-1, and macrophage inflammatory protein-1alpha); inducible nitric oxide synthase; and cyclooxygenase-2 in correspondence with the down-regulation of the nuclear factor-kappa B and mitogen-activated protein kinases signal pathways in the spinal cords from EAE mice. Interestingly, the protective effect of the SHSST was related to a decreased number of Th1 cells and an increased number of Treg cells in spinal cords from EAE mice. Taken together, our finding firstly suggested that SHSST could delay or mitigate EAE with a wide therapeutic time-window by suppressing Th1 cell responses and upregulating Treg cell responses. Also, our findings are strong enough to warrant further investigation of SHSST as a treatment for chronic autoimmune diseases including MS.

## Introduction

Multiple sclerosis (MS) is a chronic and demyelinating inflammatory disorder of the central nervous system (CNS), which typically presents in adults who are 20 to 45 years of age (Frohman et al., 2006; Axisa and Hafler, 2016). Its typical neurologic symptoms are characterized as being quadriplegia, dyschezia, weakness, amblyopia, and ataxia (Frohman et al., 2006; Axisa and Hafler, 2016). Until recently, the recruitment and infiltration of T helper type 1 (Th1) and Th17 cells into the CNS from the periphery were thought to be the main effector T cells responsible for the autoimmune demyelination (McFarland and Martin, 2007; Hoglund and Maghazachi, 2014). MS patients still get drugs to combat relapses and to slow the disease: anti-inflammatory medications (corticosteroids), interferon and interferon-beta, mitoxantrone, natalizumab, an alternative treatment method, such as acupuncture, bee sting, etc. (Mahdavian et al., 2010; Axisa and Hafler, 2016). However, they have limited benefits and disabling adverse effects, such as influenza-like syndrome and self-limited feeling, associated with long-term therapy (Neilley et al., 1996; Gasperini and Ruggieri, 2009; Minagar, 2013; Finkelsztejn, 2014). Therefore, the development of safe and efficient drugs for the treatment of the disorder – whether to delay the onset of MS or to forestall its progression is critical. Although the mechanism responsible for demyelination has been under intense study, the complete pathogenesis of MS and innovative medication for MS remain to be determined.

Recently, Oriental traditional medicines are becoming increasingly more popular because of the medicines’ beneficial efficacy in physical strength and/or disease treatment. Samhwangsasim-tang (SHSST; San-Huang-Xie-Xin-Tang in Chinese and San’o-shashin-to in Japanese) is a traditional herbal medicinal formula containing Coptidis rhizome (rhizomes of *Coptis chinensis* FRANCH), Rhei Rhizoma (rhizomes of *Rheum officinale* BAILL), and Scutellariae Radix (roots of *Scutellaria baicalensis* GEORGI) (Hur, 1613). SHSST has traditionally been used in Korea, China, and Japan as a curative for both hypertension and its complications; and it is still widely used for both purposes (Hur, 1613; Jin et al., 2007). According to previous laboratory and clinical studies, SHSST plays a role in the treatment of various diseases including hypertension (Iijima et al., 2000; Lo et al., 2005b; Jin et al., 2007), cardiac disorder (Liou et al., 2012), gastric inflammatory symptoms (Shih et al., 2007), acute lung injury (Lo et al., 2005a), and hepatic disorder (Huang et al., 2011). SHSST also exerts a neuroprotective effect in 6-OHDA-induced toxicity in neuronal SH-SY5Y cells (Shih et al., 2011) and in the MPP(+)/MPTP-induced toxicity, *in vitro* and *in vivo* model for Parkinson’s disease (Lo et al., 2012) via its anti-inflammatory, anti-oxidative, and anti-apoptotic activities. The protective effects may be associated with regulation of the nuclear factor-kappa B (NF-κB) and mitogen-activated protein kinases pathways (Shih et al., 2011; Wu et al., 2016) and regulation of Th1/Th2 T-cell balance (Li et al., 2010). These reports strongly suggest that SHSST can exert a beneficial effect in an immunological neural disorder. Therefore, we investigated the effect of SHSST on neurological symptoms and its cellular biology of myelin oligodendrocyte glycoprotein-induced experimental autoimmune encephalomyelitis (MOG-EAE) mice. We firstly found that SHSST delays or alleviates the severity of EAE by suppressing Th1 cell responses and upregulating Treg cell responses.

## Materials and Methods

### Animals

Female adult C57BL/6 mice (Narabiotec Co., Ltd., Seoul, South Korea; weight, 20.5 ± 0.5 g; Seed mice were originated from Taconic Biosciences Inc., Cambridge, IN, USA) were kept at a constant temperature of 23 ± 2°C with a 12-h light-dark cycle (light on 7:00 to 19:00), and fed food and water *ad libitum*. The animals were allowed to habituate to the housing facilities for 1 week before the experiments.

### Ethics Statement

All experimental procedures were reviewed and approved by the Institutional Animal Care and Use Committee at Kyung Hee University. In this process, proper randomization of laboratory animals and handling of data were performed in a blinded manner in accordance with recent recommendations from a NIH Workshop on preclinical models of neurological diseases (Landis et al., 2012).

### Preparation of SHSST Extracts

Three dried medicinal herbs (Coptidis rhizome, Rhei Rhizoma, and Scutellariae Radix), components of SHSST (Hur, 1613), were purchased from Omniherb Co., Ltd. (Daegu, South Korea). The SHSST extracts was prepared by previous described (Choi et al., 2015). Briefly, each dried herb was mixed in a ratio of 8:8:4 by weight (100 g in total) and cut into small pieces. The mixture was incubated in 1.0 L distilled water using a reflux extraction system for 90 min and was boiled for 90 min. The aqueous extract was filtered through Whatman No. 4 filter paper having a pore size of 20–25 μm and it was concentrated by vacuum evaporation using a EYELA N-1200A (EYELA, Rikakikai Co. Ltd., Tokyo, Japan) at 60°C. The viscous extract was lyophilized and stored at -80°C until use. The final yields were 18.3%. Total daily dose of SHSST for mice was determined using published formula for dose translation based on body surface area (Reagan-Shaw et al., 2008) after considering body weight of animal, final extract yield, and traditional dose in humans. Traditionally, human is known to drink decocted dose of dried SHSST (20 g) twice a day (Hur, 1613).

### Identification of SHSST Extract by Qualitative HPLC Analysis

Samhwangsasim-tang extract was identified using high-performance liquid chromatography by previous described (Choi et al., 2015). Briefly, a 1 g of SHSST extract was dissolved in 200 ml of 70% methanol and filtered through a 0.45 μm polyvinylidene difluoride filter. The standard materials used for the qualitative analysis of SHSST were berberine (from Coptidis rhizome), sennoside A (from Rhei Rhizoma), and baicalin (from Scutellariae Radix). The standard stock solutions were prepared by dissolving 5 mg samples, each in 50 ml methanol. The samples were separated on a Zorbax Eclipse Plus C18 column (2.1 mm × 100 mm, 1.8 μm particle size; Agilent Technology, Santa Clara, CA, USA) and the separation temperature was 25°C. The mobile phase consisted of acetonitrile (A) and 15 mM potassium dihydrogen phosphate in distilled water (B) at a mix ratio of A:B = 30:70 (v/v) with a flow rate of 0.5 mL/min. The column eluent was monitored by UV at 245 nm and 3 μl of each solvent was analyzed for 15 min.

### Experimental Group, Induction of EAE, and Clinical Evaluation

**FIGURE 1:**
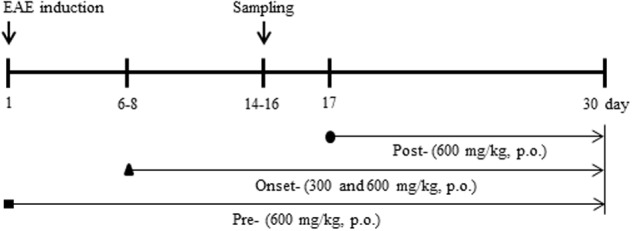
**Schematic design of the experimental protocol**. Experiments were accomplished by administering pre-treatment (daily, beginning 1 h before immunization), onset-treatment (daily, beginning approximately 6–8 days following immunization), and post-treatment (daily, beginning 16–18 days following immunization) dosages of SHSST (300 or 600 mg/kg, p.o.). A behavioral test was evaluated at the same time of day, for 30 days following immunization. Spinal cords were sampled within 14–16 days after immunization.

The experiment was accomplished by means of pre-treatment, onset-treatment, and post-treatment with SHSST (**Figure 1**). Basically, the experimental group was subdivided into the following groups (*n* = 3–6 per group): The sham group [vehicle treatment, s.c. + saline, p.o.], EAE [200 μg of MOG_35-55_, s.c. + saline, p.o.], EAE + SHSST group [200 μg of MOG_35-55_, s.c. + 300 or 600 mg/kg of SHSST, p.o.], and SHSST alone group [vehicle treatment, s.c. + 600 mg/kg of SHSST, p.o.]. EAE was induced by previous described (Choi et al., 2015; Lee et al., 2016c). Briefly, mice were immunized subcutaneously with 100 μl of an emulsion containing 200 μg of MOG_35-55_ (Sigma-Aldrich, St. Louis, MO, USA), complete Freund’s adjuvant (Sigma-Aldrich), and 550 μg of *Mycobacterium tuberculosis* H37Ra (Difco, Detroit, MI, USA) into the hind flanks. Mice were injected intraperitoneally with 250 ng of pertussis toxin (PTX; Sigma-Aldrich) on day 0 of immunization and day 2 after immunization. Mice in the sham group were treated with saline alone instead of MOG_35-55_ peptide or PTX. Clinical signs of EAE were scored daily using the clinical scoring scale as previously described (Choi et al., 2015; Lee et al., 2016c): grade 0, absence of symptoms; grade 1, partial loss of tail tonus; grade 2, paralysis of tail; grade 3, paraparesis; grade 4, paraplegia; grade 5, tetraparesis; grade 6, tetraplegia; grade 7, death.

### Histopathological Evaluation

At the peak days (14–16 days) of clinical score after induction of EAE, mice were deeply anesthetized, perfused intracardially with 0.9% saline followed by 4% paraformaldehyde (PFA) in 0.1 M phosphate buffer (PB, pH 7.4). Lumbar spinal cords were removed, fixed using 4% PFA for a day, incubated overnight in 30% sucrose solution, and then cut into 10-μm thick by previously described (Lee et al., 2016a,b). The sections were stained with luxol fast blue (LFB) and counterstained by hematoxylin to evaluate demyelination and immune cell infiltration, respectively. The sections were dehydrated and coverslipped as previously described (Choi et al., 2015; Lee et al., 2016c). The level of demyelination after LFB staining was evaluated as previously described (Fissolo et al., 2012): 0, no demyelination; 1, little demyelination, only around infiltrates and involving less than 25% of the white matter; 2, demyelination involving less than 50% of the white matter; 3, diffuse and widespread demyelination involving more than 50% of the white matter. The level of recruitment/infiltration of immune cells after hematoxylin staining was scored according to the following criteria (Fissolo et al., 2012): 0, no lesion; 1, cellular recruitment/infiltration only in the meninges; 2, very discrete and superficial infiltrates in parenchyma; 3, moderate infiltrate (less than 25%) in the white matter; 4, severe infiltrates (less than 50%) in the white matter; 5, more severe infiltrates (more than 50%) in the white matter.

### Immunohistochemistry

At the peak stage (14–16 days) of clinical score after induction of EAE, spinal sections were immunostained as previously described (Choi et al., 2015; Lee et al., 2016a,b,c) using rabbit anti-ionized calcium binding adaptor molecule-1 (Iba-1) (1:2,000; WAKO, Osaka, Japan) or rabbit anti-glial fibrillary acidic protein (GFAP) (1:2,000; DACO, USA) as the primary antiserum, biotinylated rabbit IgG antibody (1:200; Vector Laboratories, USA) as the secondary antiserum, avidin-biotinylated horseradish peroxidase-complex (1:200; Vector Laboratories), and 3,3′-diamino-benzidine.

### Real Time Polymerase Chain Reaction (RT-PCR)

At the peak stage (14–16 days) of clinical score after induction of EAE, the lumbar segments of the spinal cords were harvested and total RNA was extracted from spinal cord using TRIsure reagent according to the manufacturer’s instructions (Bioline, UK). cDNA was synthesized by incubating 1 μg of total RNA for 1 h at 37°C in a reaction mixture containing 0.5 μg of Oligo dT, 0.5 mM dNTP mix, 5x first-strand buffer, RNase out, 5 mM dithiothreitol (DTT), and M-MLV reverse transcriptase. Real-time PCR was accomplished using SYBR Green PCR Master Mix (Applied Biosystems, Franklin Lakes, NJ, USA) as previously described (Choi et al., 2015; Lee et al., 2016a,b,c). Fold-induction was calculated using the 2^-ΔΔCT^ method. PCR amplification was performed at least three times. Sequences of oligonucleotide primers were listed in **Table 1**.

**Table 1:** PCR primer sequence for PCR analysis.

Primer		Primer sequence (5′→3′)
CD11b	Forward Reverse	TGC TTA CCT GGG TTA TGC TTC TG CCG AGG TGC TCC TAA AAC CA
CD3	Forward Reverse	CTC TGG GCT TGC TGA TGG GGT TGG GAA CAG GTG GTG
Claudin-3	Forward Reverse	CTG GGA GGG CCT GTG GAT GAA CT TCG CGG CGC AGA ATA GAG GAT
Claudin-5	Forward Reverse	ACG GGA GGA GCG CTT TAC GTT GGC GAA CCA GCA GAG
COX-2	Forward Reverse	GCT CGG CTT CCA GTA TTG AG AGA AGG AAA TGG CTG CAG AA
Foxp3	Forward Reverse	GGC CCT TCT CCA GGA CAG A GCT GAT CAT GGC TGG GTT GT
GAPDH	Forward Reverse	AGG TCA TCC CAG AGC TGA ACG CAC CCT GTT GCT GTA GCC GTA T
ICAM-1	Forward Reverse	TGC GTT TTG GAG CTA GCG GAC CA CGA GGA CCA TAC AGC ACG TGC AG
IFN-γ	Forward Reverse	ACA ATG AAC GCT ACA CAC TGC AT TGG CAG TAA CAG CCA GAA ACA
IL-1β	Forward Reverse	TTG TGG CTG TGG AGA AGC TGT AAC GTC ACA CAC CAG CAG GTT
IL-2	Forward Reverse	GCC CAA GAA GGC CAC AGA GCA CTT CCT CCA GAG GTT TGA G
IL-4	Forward Reverse	CGA AGA ACA CCA CAG AGA GTG AGC T GAC TCA TTC ATG GTG CAG CTT ATC G
IL-5	Forward Reverse	GAG TCA TGA GAA GGA TGC TT ACA GTT TTG TGG GGT TTT TG
IL-10	Forward Reverse	ATA ACT GCA CCC ACT TCC CA TCA TTT CCG ATA AGG CTT GG
IL-17	Forward Reverse	GTG TCT CTG ATG CTG TTG AAC GGT TGA GGT AGT CTG
IL-23	Forward Reverse	TGG CAT CGA GAA ACT GTG AGA TCA GTT ATT GGT AGTCCT GTT
iNOS	Forward Reverse	GGC AAA CCC AAG GTC TAG GTT TCG CTC AAG TTC AGC TTG GT
MBP	Forward Reverse	CTA TAA ATC GGC TCA CAA GG AGG CGG TTA TAT TAA GAA GC
MCP-1	Forward Reverse	CTT CTG GGC CTG CTG TTC A CCA GCC TAC TCA TTG GGA TCA
MIP-1α	Forward Reverse	CAG CCA GGT GTC ATT TTC CT AGG CAT TCA GTT CCA GGT CA
PDGFαR	Forward Reverse	GCC AGG AGA CGA GGT ATC AA TGT TCC CAA TGC CAA GGT C
RANTES	Forward Reverse	ACA CCA CTC CCT GCT GCT TT GAC TGC AAG ATT GGA GCA CTT G
TGF-β	Forward Reverse	GCC CTG GAT ACC AAC TAT TGC GCA GGA GCG CAC AAT CAT GTT
TNF-α	Forward Reverse	AGC AAA CCA CCA AGT GGA GGA GCT GGC ACC ACT AGT TGG TTG T
VCAM-1	Forward Reverse	CCT CAC TTG CAG CAC TAC GGG CT TTT TCC AAT ATC CTC AAT GAC GGG
ZO-1	Forward Reverse	AAG GCA ATT CCG TAT CGT TG CCA CAG CTG AAG GAC TCA CA

### Western Blot Analysis

At the peak stage (14–16 days) of clinical score after induction of EAE, the lumbar segments of the spinal cords were harvested. Western blot analysis was performed as previously described (Jang and Cho, 2016; Lee et al., 2016a) using rabbit anti-p-IκBα (1:500; Santa Cruz Biotechnology, Santa Cruz, CA, USA), rabbit anti-NF-κB p65 (1:1,000; Santa Cruz Biotechnology), rabbit anti-p-ERK/p-JNK/p-P38 (1:1,000; Cell Signaling Technology, Beverly, MA, USA), rabbit anti-ERK/JNK/P38 (1:1,000; Cell Signaling Technology), goat anti-IL-10 (1:500, R&D systems, Minneapolis, MN, USA), rabbit anti-inducible nitric oxide synthases (iNOS) (1:500; sigma), and mouse anti-cyclooxygenase-2 (COX-2) (1:500, BD Biosciences, San Joes, CA, USA) antibodies, and HRP-conjugated secondary antibodies.

### Flow Cytometry

For flow cytometry analysis, six mice in each group at peak period (day 14–16) after induction of EAE were anesthetized and lumbar segments of spinal cords were carefully dissected and dissociated as previously described (Choi et al., 2015; Lee et al., 2016a,b,c). Briefly, single-cell suspensions refined from whole tissue were prepared (centrifuged at 300 g for 5 min) and fixed with 2% PFA, cells were washed with washing buffer containing 2% fetal bovine serum (FBS) in PBS, incubated with mouse anti-mouse CD32 (BD Bioscience) for 10 min to block the Fc receptor and washed twice with 2% FBS washing buffer. For cell surface staining of immune markers with fluorescently labeling, the cells were incubated with APC anti-mouse CD4 (RM4-5, BD Biosciences), PE anti-mouse CD8a (53-6.7, BD Biosciences), APC anti-mouse/human CD11b (M1/70; BioLegend), and PE anti-mouse CD45 (30-F11; BD Biosciences) for 30 min at 4°C. Non-stained cells were used as negative controls. For intracellular cell staining, cells were restimulated with PMA (phorbol 12-myristate-13-acetate, Sigma), ionomycin (Sigma), and Golgistop (protein transport inhibitor, BD Biosciences) for 5 h at 37°C incubator. After this stimulation, cells were fluorescent stained with PerC3 anti-mouse CD4 (RM4-5, BD Biosciences), FITC anti-mouse IFN-γ (BD Biosciences), PE anti-mouse IL-17A (TC11-18H10, BD Biosciences), PE anti-mouse IL-4 (11B11; BioLegend), APC anti-mouse/rat forkhead box P3 (Foxp3) (FJK-16s; eBioscience) for 30 min. The stained cells were washed twice with 2% FBS washing buffer and used for flow cytometry. Data were collected on a FACSCalibur flow cytometer (BD Biosciences) and analyzed using CellQuest Pro software (BD Biosciences). CD4^+^ T cells were gated to analyze populations of Th1, Th2, Th17, and Treg cells. Three-color staining of one cell for simultaneous analysis and results of intracellular cytokines were indicated as the percentage within the CD4^+^ population. We acquired through set up of 10,000 cells events, and gating was set by side scatter.

### Statistical Analysis

Statistical analysis was performed by using the SPSS 21.0 package (SPSS Inc, Chicago, IL, USA) for Windows. All of the data were presented as mean ± SEM. Sum of neurological score, histological score, and immunological assays were compared using the one-way ANOVA with Tukey *post hoc* test for comparison of multiple groups. The *P*-values of less than 0.05 were accepted as statistically significant.

## Results

### Effect of SHSST on the Neurological Symptoms of EAE Mice

**FIGURE 2:**
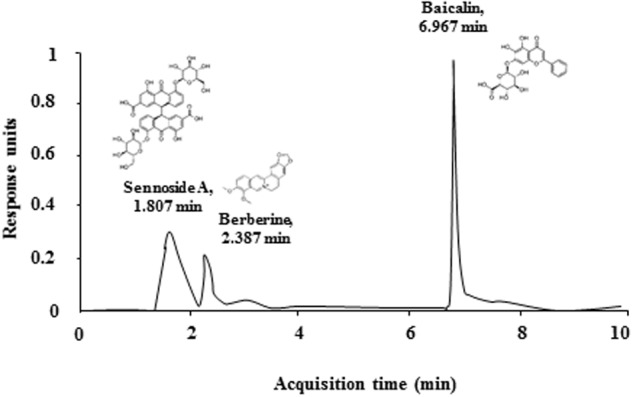
**Quantitative HPLC analysis of the contents of SHSST**. Peaks for berberine (of Coptidis rhizome), sennoside A (of Rhei Rhizoma), and baicalin (of Scutellariae Radix) corresponded to standard at 2.387, 1.807, and 6.967 min, respectively.

**FIGURE 3:**
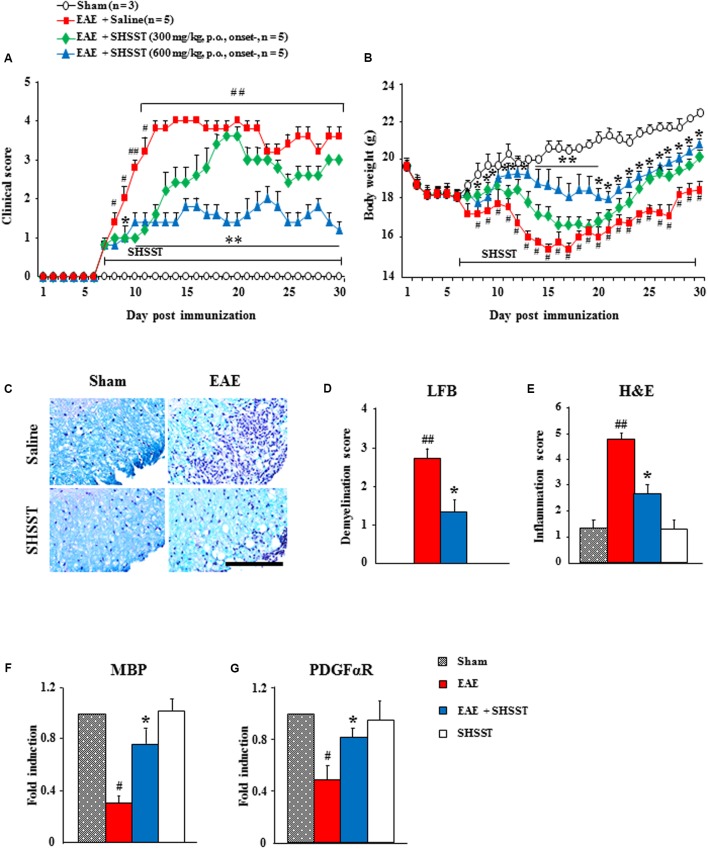
**The effect of onset-treatment with SHSST on clinical symptoms and demyelination of EAE mice. (A,B)** Following immunization, the clinical signs of mice from sham, EAE, EAE + SHSST, and SHSST groups were scored daily **(A)** and weighed daily **(B)**, until 30 days had passed. SHSST (300 or 600 mg/kg) was orally administrated daily, beginning from the onset stage (day 6–8 after immunization) of clinical signs of EAE. **(C–E)** Lumbar spinal cord sections were obtained from each group at day 14–16 post-immunization. The sections were stained with luxol fast blue and counterstained by hematoxylin **(C)**; and the levels of demyelination **(D)** and inflammation **(E)** were quantified. **(F,G)** Lysate of the lumbar spinal cord, obtained from each group at day 14–16 post-immunization, was analyzed for mRNA expression of MBP **(F)** and PDGFαR **(G)** by real-time PCR. Bar = 10 μm. Each of the quantified data are expressed as mean clinical scores, body weight, demyelination score, infiltration score, or fold induction ±SEM. (Student *t*-tests were performed for A and B; and ANOVA testing was performed; ^#^*p* < 0.05 and ^##^*p* < 0.01 versus the sham group; ^∗^*p* < 0.05 and ^∗∗^*p* < 0.01 versus the EAE group.)

First, the qualitative determination of water-extracted SHSST was performed by HPLC. Peaks for berberine (from Coptidis rhizome), sennoside A (from Rhei Rhizoma), and baicalin (from Scutellariae Radix) corresponded to standard at 2.387, 1.807, and 6.967 min, respectively. The final concentration for each constituent was 0.3 g/ml, 0.4 g/ml, and 1.1 g/ml, respectively (**Figure 2**). Consequently, we tested whether onset-treatment with SHSST could alleviate the clinical signs of EAE (**Figure 3** and **Table 2**). All mice who had undergone induction of EAE began to display early clinical signs of the disease (partial loss of tail tonus, 0.5) from 7.2 ± 0.2 days after induction of EAE. The mean clinical score in EAE group gradually increased at 7–12 days after immunization, peaked at 14–16 days (4.0 ± 0.0), and maintained thereafter. However, the mean clinical scores in EAE + SHSST (300 and 600 mg/kg) group were relatively low at 8–30 days after immunization, as compared to the EAE group (**Figures 3A,B** and **Table 2**). Mean body weight was found to have decreased in inverse proportion to the clinical score in mice from the EAE group, while the trend toward declining body weight was significantly blocked in mice from EAE + SHSST group, as compared to EAE group (**Figures 3A,B**). Treatment with SHSST alone did not affect the clinical score and weight gain of normal mice (**Figures 3A,B** and **Table 2**). Our findings suggest that onset-treatment with SHSST may alleviate both the development and the progression of EAE.

**Table 2:** Effect of onset-treatment with SHSST on a subsequent encephalogenic challenge.

	Group	Incidence (%)	Mean day of onset (±SEM)	Maximal clinical score (±SEM)	Sum of clinical score (±SEM)	Mortality (%)
Onset-	Sham	3/3	0.0 ± 0.0	0.0 ± 0.0	0.0 ± 0.0	0
	EAE	5/5	7.2 ± 0.2	4.0 ± 0.0^##^	80.2 ± 0.8^##^	0
	EAE + SHSST 300 mg/kg	5/5	7.2 ± 0.2	3.6 ± 0.2	57.6 ± 2.0^∗^	0
	EAE + SHSST 600 mg/kg	5/5	7.6 ± 0.6	2.2 ± 0.2^∗^	35.2 ± 1.9^∗∗^	0
Pre-	Sham	3/3	0.0 ± 0.0	0.0 ± 0.0	0.0 ± 0.0	0
	EAE	6/6	7.8 ± 0.3	4.0 ± 0.0^##^	73.3 ± 1.4^##^	0
	EAE + SHSST 600 mg/kg	6/6	10.8 ± 1.0	2.7 ± 0.2^∗^	40.8 ± 2.6^∗^	0
Post-	Sham	3/3	0.0 ± 0.0	0.0 ± 0.0	0.0 ± 0.0	0
	EAE	6/6	8.2 ± 0.5	3.5 ± 0.2^#^	58.7 ± 3.2^##^	0
	EAE + SHSST 600 mg/kg	6/6	8.2 ± 0.5	3.5 ± 0.2	56.7 ± 2.2	0

*Data are expressed as mean ± SEM. (ANOVA testing was performed; ^#^*p* < 0.05 and ^##^*p* < 0.01 versus the sham group; ^∗^*p* < 0.05 and ^∗∗^*p* < 0.01 versus the EAE group.)*

### Effect of SHSST on Demyelination and Infiltration of Inflammatory Cells in the Spinal Cords from EAE Mice

Since spinal demyelination is a typical histopathological characteristic of MS patients (Frohman et al., 2006; Axisa and Hafler, 2016), we tested the correlation between clinical score of EAE and demyelination. And since onset-treatment with 600 mg/kg SHSST was more effective than was 300 mg/kg SHSST in inhibiting EAE symptoms (**Figures 3A,B** and **Table 2**), we further studied use of that dosage (600 mg/kg). The sections of lumbar spinal cord at 14–16 days after the immunization (the peak period of clinical scores) were stained by LFB dye and counterstained by hematoxylin (**Figure 3C**); and were quantified (**Figures 3D,E**). The level of demyelination (pale portion) was the highest in the spinal white matter of mice in the EAE group, while the level was significantly lower in the EAE + SHSST group as compared to the EAE group (**Figures 3C–E**), which is in inverse proportion to the level of infiltration of inflammatory cells (blue colored dots) (**Figures 3C–E**). According to the immunostaining, using myelin basic protein (MBP) antibody (marker for myelin), MBP-immunoreactivity was significantly reduced in the EAE group, whereas the reduction was alleviated in the EAE + SHSST group (**Figures 3F,G**) in agreement with the alteration of the mRNA expression of MBP and alpha receptor for platelet-derived growth factor (PDGFαR, marker for oligodendrocyte), as shown by real-time PCR analysis (**Figures 3F,G**). The SHSST itself did not induce demyelination or alteration of the myelin molecules.

### Effects of SHSST on the Recruitment and Infiltration of Myeloid Cells in the Spinal Cords from EAE Mice

**FIGURE 4:**
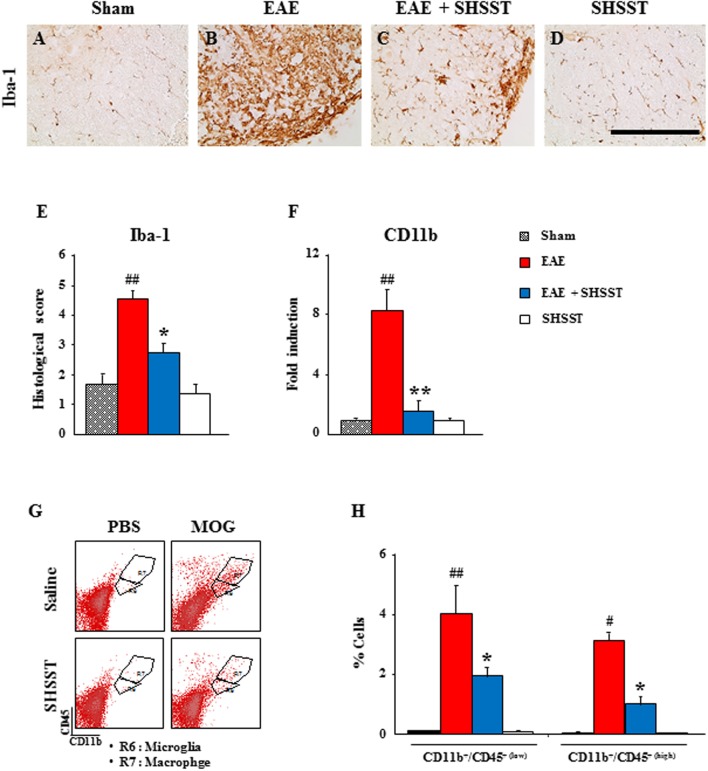
**The effect of onset-treatment with SHSST on recruitment/infiltration of residential microglia and peripheral macrophages in the spinal cords from EAE mice. (A–E)** Lumbar spinal cord sections were obtained from the sham, EAE, EAE + SHSST, and SHSST groups at day 14–16 post-immunization. The sections were immunostained with anti-Iba-1 antiserum **(A–D)**, and their immunoreactivity was quantified **(E). (F)** Lysate of the lumbar spinal cord, obtained from each group at day 14–16 post-immunization, was analyzed for mRNA expression of CD11b by real-time PCR. **(G,H)** Lumbar spinal cords were dissected from each group at day 14–16 post-immunization and analyzed to investigate the degree of recruitment/infiltration of microglia and macrophages by flow cytometry **(G)**; and were quantified **(H)**. CD11b^+^ cells were divided into CD11b^+^/CD45^+(low)^ cells (R6; microglia) and CD11b^+^/CD45^+(high)^ cells (R7; macrophage) populations **(G)**, and the percentages of each population are denoted in the graph **(H)**. Bars = 10 μm. Quantified data are expressed as mean inflammation scores, fold induction or % cells ±SEM. (ANOVA testing was performed; ^#^*p* < 0.05 and ^##^*p* < 0.01 versus the sham group; ^∗^*p* < 0.05 and ^∗∗^*p* < 0.01 versus the EAE group.)

Since mononuclear cells (resident microglia and peripheral macrophages) are recruited/infiltrated into demyelinated lesions in MS patients and EAE models (Rawji and Yong, 2013; Hoglund and Maghazachi, 2014), we tested the regulating effect of SHSST on the recruitment/infiltration of mononuclear cells into the spinal cords from EAE mice. Iba-1 (a marker for microglia/macrophage lineage cells)-immunoreactive cells displayed an activated form of enlarged cell bodies with short and thick processes in the spinal cords from the EAE group (**Figures 4B,E**), while the activation of the cells was significantly inhibited in the EAE + SHSST group (**Figures 4C,E**). In agreement with morphological changes of Iba-1-immunoreactive cells, mRNA expression of CD11b was up-regulated in the EAE group, as compared to the sham group, whereas the elevated expression of CD11b was significantly diminished in the EAE + SHSST group (**Figure 4F**). SHSST itself did not induce activation of Iba-1-immunoreactive cells (**Figures 4A–E**) and mRNA expression of CD11b as compared to the sham group (**Figure 4F**). Here, since Iba-1-immunoreactivity displays both resident microglia and peripheral macrophages (Imai et al., 1996), the population of these two cells was differentiated by flow cytometry (**Figures 4G,H**). The populations of CD11b^+^/CD45^+(low)^ cells representing the microglia population and CD11b^+^/CD45^+(high)^ cells representing the macrophages (Campanella et al., 2002; Lee et al., 2016a,b,c) were increased in the spinal cords from the EAE group (4.0 ± 0.9 and 3.1 ± 0.3%, respectively), as compared to the sham group (0.1 ± 0.0 and 0.1 ± 0.0%, respectively); while these increased population was inhibited in the EAE + SHSST group (1.9 ± 0.3 and 1.0 ± 0.2%, respectively), as compared to the EAE group (**Figures 4G,H**). The results indicate that the onset-treatment with SHSST could reduce clinical signs of EAE by the inhibiting of the activation/infiltration of residential microglia and peripheral macrophages.

### Effects of SHSST on BBB Integrity in the Spinal Cords from EAE Mice

**FIGURE 5:**
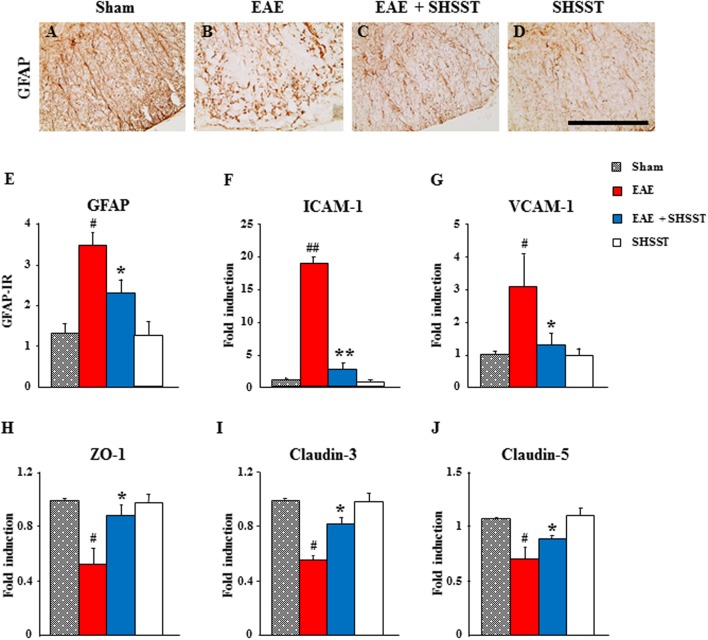
**The effect of onset-treatment with SHSST on BBB integrity in the spinal cords from EAE mice. (A–E)** Lumbar spinal cord sections were prepared from sham, EAE, EAE + SHSST, and SHSST groups at day 14–16 post-immunization. Sections were immunostained with anti-GFAP antiserum **(A–D)**, and their immunoreactivity was quantified **(E). (F–J)** Lysates of spinal cords from each group were analyzed for mRNA expression of ICAM-1 **(F)**, VCAM-1 **(G)**, ZO-1 **(H)**, claudin-3 **(I)**, and claudin-5 **(J)** by real-time PCR. Bars = 10 μm. Quantified data are expressed as mean immunoreactivity or fold induction ±SEM. (ANOVA testing was performed; ^#^*p* < 0.05 and ^##^*p* < 0.01 versus the sham group; ^∗^*p* < 0.05 and ^∗∗^*p* < 0.01 versus the EAE group.)

Since astrocytes are essential for the formation and maintenance of the blood–brain barrier (BBB) (Willis, 2010), we used immunohistochemistry to test the level of astroglial activation in the spinal cords from EAE mice. The intensity of GFAP-immunoreactive astrocytes was increased in the spinal cords from the EAE group, whereas the increase in intensity was inhibited in the EAE + SHSST group (**Figures 5A–E**). Since the permeability of BBB was mediated by alteration of adhesion and junctional molecules, we used real-time PCR analysis to test the effect of SHSST on their expression. The mRNA expressions of endothelial ICAM-1 and VCAM-1 were up-regulated in the spinal cords from mice in the EAE group, while the up-regulations were inhibited in the EAE + SHSST group (**Figures 5F,G**). Also, the mRNA expressions of zona occludens-1, claudin-3, and claudin-5 were down-regulated in the spinal cords from mice in the EAE group, while the down-regulations were inhibited in the EAE + SHSST group (**Figures 5H–J**). Our findings indicates that onset-treatment with SHSST may exert a protective effect on EAE mice through inhibiting astroglial activation and decreasing or increasing expressions of adhesive and junctional molecules associated with a reduction in BBB permeability.

### Effects of SHSST on Infiltration of CD4^+^ T Cells in the Spinal Cords from EAE Mice

**FIGURE 6:**
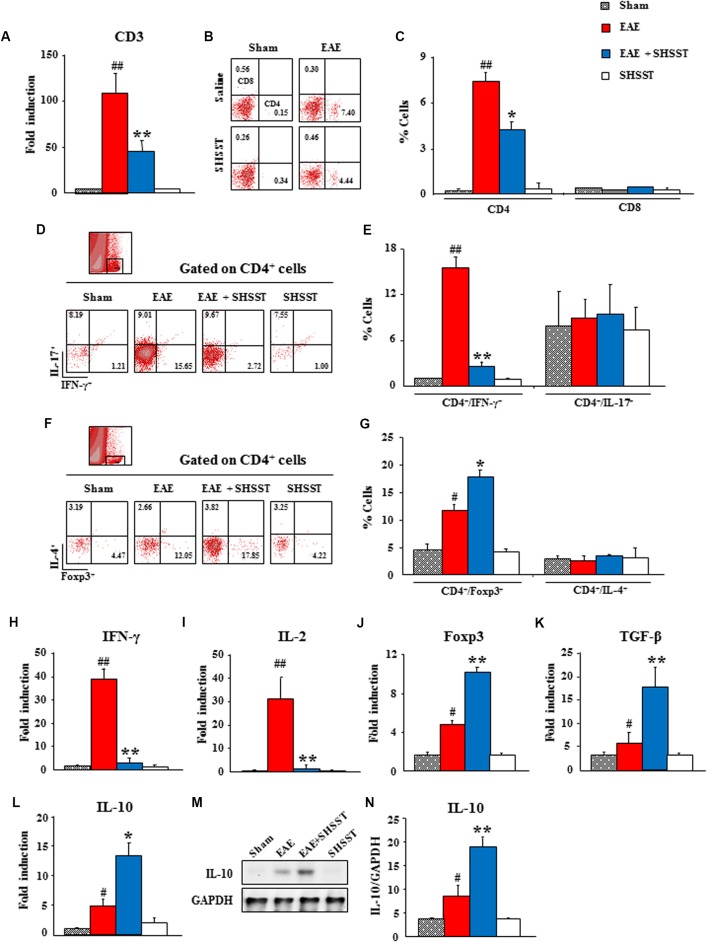
**The effect of onset-treatment with SHSST on recruitment/infiltration of CD4^+^ T cells and its subsets in the spinal cords from EAE mice. (A–G)** Lumbar spinal cord lysates were prepared from sham, EAE, EAE + SHSST, and SHSST groups at day 14–16 post-immunization. Each was analyzed for mRNA expression of CD3 by real-time PCR **(A)** and analyzed by flow cytometry **(B–G)**. The population of CD4^+^ and CD8^+^ **(B,C)**, CD4^+^/IFN-γ^+^ and CD4^+^/IL-17^+^ **(D,E)**, and CD4^+^/Foxp3^+^ and CD4^+^/IL-4^+^ T cells **(F,G)** were dotted and displayed in graphs. R10 **(D)** and R8 **(F)** were gated as CD4^+^ T cells. **(H–N)** Lumbar spinal cord lysates were prepared from each group and analyzed for mRNA expression of IFN-γ **(H)**, IL-2 **(I)**, Foxp3 **(J)**, TGF-ß **(K)**, and IL-10 **(L)** by real-time PCR and protein expression of IL-10 **(M)** by immunoblotting **(M,N)**. Quantified data are expressed as mean % cells, fold induction, or protein expression ±SEM. (ANOVA testing was performed; ^#^*p* < 0.05 and ^##^*p* < 0.01 versus the sham group; ^∗^*p* < 0.05 and ^∗∗^*p* < 0.01 versus the EAE group.)

Since auto-reactive T cells were recruited/infiltrated into the lesions of CNS through the BBB from the periphery (Hohlfeld, 1997), we tested whether SHSST might regulate the differentiation of T cells in the periphery and their recruitment/infiltration into EAE mice’s CNS. By real-time PCR analysis, the mRNA expression of CD3, a marker for T cell, was found to have significantly increased in the spinal cords from EAE mice, as compared to the sham group; while the expression of this marker was significantly low in the EAE + SHSST group, as compared to the EAE group (**Figure 6A**), in agreement with the level of clinical score and spinal demyelination (**Figures 3A–G**). Consecutively, the populations of helper T (Th; CD4^+^) cells and cytotoxic (CD8^+^) T cells were differentiated by cytometry. The populations of CD4^+^ T cells were found to be increased in the spinal cords (7.4 ± 0.5%) from EAE mice, as compared to the sham group (0.2 ± 0.0%); whereas the populations of such cells were significantly decreased in the EAE + SHSST group (4.4 ± 0.7%), as compared to the EAE group. However, the population of CD8^+^ T cells was not significantly affected either by immunization or by the onset-treatment of SHSST (**Figures 6B,C**).

Since an imbalance in the subsets of CD4^+^ T cells was found in the EAE model and MS patients (Dittel, 2008; Hoglund and Maghazachi, 2014; Dendrou et al., 2015), we investigated the population of Th1 (CD4^+^/IFN-γ^+^), Th2 (CD4^+^/IL-4^+^), Th17 (CD4^+^/IL-17^+^), and Treg (CD4^+^/Foxp3^+^) cells in the spinal cord. First, the population of CD4^+^/IFN-γ^+^ T cells was significantly increased in spinal cords from mice in the EAE group (15.7 ± 1.4%), as compared to the sham group (1.2 ± 0.0%); while the population of such cells was significantly decreased in the EAE + SHSST group (2.7 ± 0.6%) (**Figures 6D,E**), corresponding to the alteration in the level of mRNA expression of IFN-γ and IL-2 (interleukins produced by Th1 cells) in the spinal cord. Fold inductions for IFN-γ in the sham, EAE, EAE + SHSST, and SHSST groups were 1.0 ± 0.0, 39.2 ± 4.0, 3.4 ± 1.9, and 1.0 ± 0.1, respectively (**Figure 6H**). And fold inductions for IL-2 were 1.0 ± 0.0, 31.5 ± 8.9, 1.7 ± 0.4, and 1.3 ± 0.3, respectively (**Figure 6I**).

The population of CD4^+^/Foxp3^+^ T cells, formerly known as suppressor T cells (Dittel, 2008; Hoglund and Maghazachi, 2014), was significantly increased in the spinal cords from EAE mice (12.0 ± 2.1%), as compared to that of the sham group (4.5 ± 1.3%). This increase was more pronounced in the EAE + SHSST group (17.9 ± 3.1%) (**Figures 6F,G**), which corresponded to the alteration of mRNA expression for Foxp3 (a key transcription factor for differentiation and function of Tregs) (**Figure 6J**) and TGF-β (an inducer of Treg cells) (**Figure 6K**) and both mRNA and protein expression levels of IL-10 (an interleukin produced by Tregs cells) (**Figures 6L–N**). However, the populations of CD4^+^/IL-17^+^ and CD4^+^/IL-4^+^ T cells was not significantly changed in the spinal cords from either EAE mice or SHSST onset-treated mice (**Figures 6D–G**). These findings are in parallel with the unchanged mRNA expressions of IL-17 (an interleukin produced by Th17 cells), IL-23 (an interleukin produced by Th17 cells), IL-4 (a cytokine that induces differentiation of naive Th cells into Th2 cells), and IL-5 (an interleukin produced by Th2 cells) in the spinal cord (**Supplementary Data S1**). The findings suggest that onset-treatment with SHSST could reduce the clinical signs of EAE by inhibiting differentiation/recruitment/infiltration of Th1 cells and increasing that of Tregs cells.

### Effects of SHSST on the Inflammatory Mediators in the Spinal Cords from EAE Mice

**FIGURE 7:**
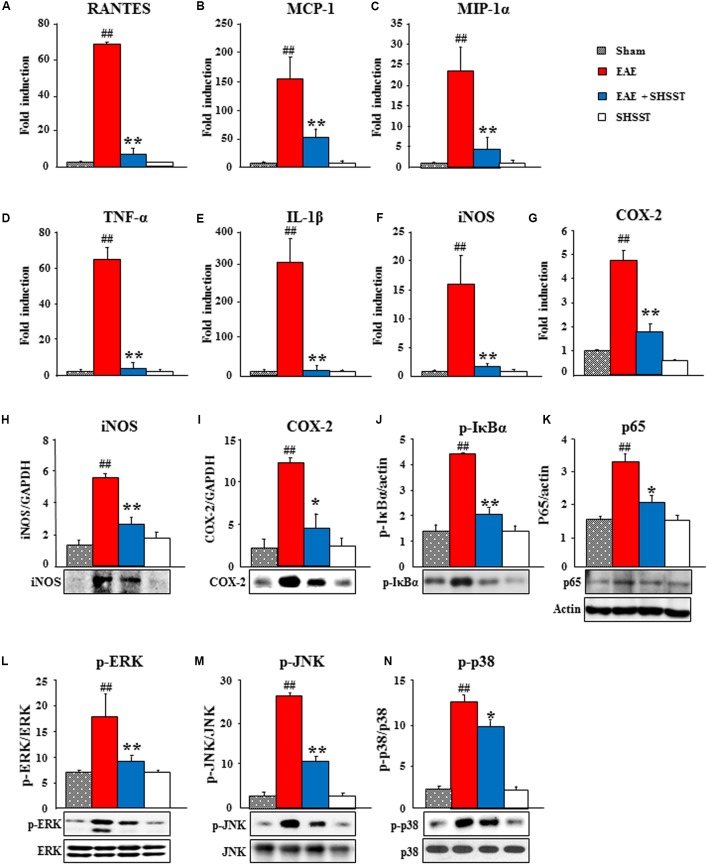
**The effect of onset-treatment with SHSST on expression of inflammatory mediators in the spinal cords from EAE mice. (A–G)** Lumbar spinal cord lysates (*n* = 5 per group) were prepared from sham, EAE, EAE + SHSST, and SHSST groups at day 14–16 post-immunization. Each was analyzed to measure the level of the mRNA expression of RANTES **(A)**, MCP-1 **(B)**, and MIP-1α **(C)** as representative chemokines, TNF-α **(D)** and IL-1ß **(E)** as representative cytokines, iNOS **(F)**, and COX-2 **(G)** with real-time PCR. **(H–N)** Lumbar spinal cord lysates from each group (*n* = 3–5 per group) were analyzed to measure the level of the protein expression of iNOS **(H)**, COX-2 **(I)**, p-IκBα **(J)**, NF-κB (p65) **(K)**, p-ERK **(L)**, p-JNK **(M)**, and p-p38 **(N)** by immunoblotting; and were quantified. Actin band in **K** was shared in **H–J**. Quantified data are expressed as mean fold induction or protein expression ±SEM. (ANOVA testing was performed; ^##^*p* < 0.01 versus the sham group; ^∗^*p* < 0.05 and ^∗∗^*p* < 0.01 versus the EAE group.)

Since inflammatory mediators, such as cytokines and chemokines, play essential roles in the immune response system by activating and recruiting microglia/macrophage and T cells during the progression of MS and EAE (Imitola et al., 2005; Szczucinski and Losy, 2007; Petermann and Korn, 2011), we tested the mRNA expression of representative inflammatory mediators in the spinal cord at 14–16 days after induction of EAE (**Figure 7**). Real-time PCR data displayed little or no mRNA expression of representative chemokines [RANTES (also known as CCL5), monocyte chemotactic protein-1 (MCP-1; also referred to as CCL2), macrophage inflammatory protein (MIP)-1α], cytokines [tumor necrosis factor-α (TNF-α) and IL-1β], iNOS, and COX-2 in spinal cords from the sham and SHSST alone groups. These expressions were remarkably increased in the EAE group, whereas the increases were significantly inhibited in the EAE + SHSST group (**Figures 7A–G**). In agreement with these results, protein expression levels of iNOS and COX-2 were significantly lower in the EAE + SHSST group compared to those in the EAE group (**Figures 7H,I**). Since NF-κB and MAPKs signaling pathways activate inflammatory response during MS and EAE (Pahan and Schmid, 2000; Brereton et al., 2009; Krementsov et al., 2013), we investigated the effect of onset-treatment with SHSST on NF-κB and MAPKs signaling pathways by Western blot analysis. Onset-treatment with SHSST significantly blocked increase in protein expression of p-IκBα, NF-κB (p65), p-ERK, p-JNK, and p-P38 in the spinal cords from EAE mice (**Figures 7J–N**). Our findings indicates that onset-treatment with SHSST may alleviate clinical-score-assessed levels of EAE via the inhibiting expression of chemokines/cytokines, iNOS, and COX-2 associated with inactivation of the NF-κB and MAPKs signaling pathways, in the spinal cords from EAE mice.

### Preventive and Therapeutic Effects of SHSST on EAE

**FIGURE 8:**
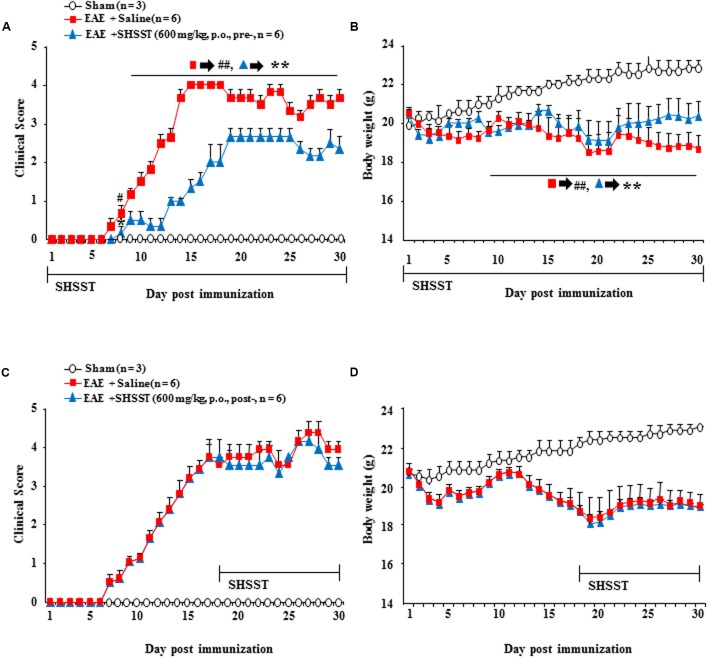
**Effect of pre-treatment and post-treatment with SHSST on clinical symptoms of EAE mice. (A–D)** SHSST (600 mg/kg) was orally administrated once daily from 1 h before immunization (pre-treatment; **A** and **B**) and peak stage (day 16–18 after immunization) of clinical sign (post-treatment; **C** and **D**) of EAE mice. After the immunization, clinical signs of all mice were scored **(A,B)**; and the mice were weighed **(C,D)** daily until 30 days later. Data are expressed as mean clinical scores ±SEM. (ANOVA testing was performed; ^##^*p* < 0.01 versus the sham group; ^∗∗^*p* < 0.01 versus the EAE group.)

We additionally investigated the preventive and therapeutic time window for clinical signs of EAE. As show in **Figures 3**–**7**, we demonstrated a therapeutic effect of SHSST and its cellular mechanism in the onset stage of clinical score. Additionally, we investigated the effect of pretreatment (a daily dose from 7 days before the induction of EAE) and post-treatment (a daily dose from 17 days after the induction of EAE; peak stage of clinical score) with SHSST in EAE mice (**Figure 8**). Pretreatment with SHSST delayed the onset of neurological impairment and alleviated EAE symptoms (**Figure 8A**), corresponding with a change of body weight (**Figure 8B**). However, the post-treatment with SHSST did not produce a remarkable positive effect on either the level of neurological impairment or in a change in body weight in the EAE mice (**Figures 8C,D**). Taken together, our findings indicate that while SHSST has preventive (**Figures 8A,B**) and therapeutic effects in the early stage of clinical signs (**Figures 3**–**7**), it does not have therapeutic effect in the late stage of clinical signs for EAE (**Figures 8C,D**).

## Discussion

Multiple sclerosis and experimental autoimmune encephalo-myelitis are characterized as chronic demyelination induced by the CNS infiltration of autoreactive T cells through the BBB from the periphery (Frohman et al., 2006; Axisa and Hafler, 2016). Based on the literature, SHSST – a traditional herbal formulation – has promising novel immunomodulatory and anti-inflammatory activities (Hur, 1613; Li et al., 2010; Shih et al., 2011), but its mechanism underlying demyelinating disorder is still unclear. Here, we have demonstrated for the first time that SHSST restrains the development and progression of EAE via its immunomodulatory and anti-inflammatory activities. Its mechanism was associated with the inhibited infiltration and the activation of microglia and macrophages, the expression of inflammatory mediators, and the maintenances of BBB integrity as well as reduced demyelination and neural damage, through inhibiting Th1 cell response and activating Treg cell response. Our findings suggest that SHSST may be an attractive candidate from which to develop an effective and safe medication and functional food for MS patients.

Herbal medicine has become a popular form of healthcare (Firenzuoli and Gori, 2007). According to the literature, SHSST has been used in East Asia as a representative herbal medicine to treat both hypertension and its complications (Hur, 1613). Recently, scientific studies have newly demonstrated that SHSST has pharmacological efficacy, due to its having anti-inflammatory, anti-oxidative stress, anti-cancer, and neuroprotective properties (Li et al., 2010; Shih et al., 2011; Liou et al., 2012; Lo et al., 2012). These properties would mainly be associated with the constituents of SHSST such as berberine, coptisine, berberastine, worenine, and palmatine from *Coptis Rhizome* (Manisha, 2009); hydroxyanthracene derivatives (emodin, physcione, aloe-emodin, and chrysophanol glycosides) from *Rhei Rhizoma* (Manisha, 2009); and aglycones (baicalein, wogonin, oroxylin A) and glycosides (baicalin, wogonoside, oroxylin A-7-glucuronide) from *Scutellariae Radix* (Li et al., 2011). Berberine was found to attenuate both the clinical and pathological parameters of EAE and the permeability of the BBB by inhibiting matrix metalloproteinase-9 and laminin degradation (Jiang et al., 2013). Baicalin and baicalein both ameliorated clinical disease severity in EAE and reduced demyelination in, immune cell infiltration into, and inflammation in the spinal cord through the inhibition of 12/15-lipoxygenase (Xu et al., 2013) and the inhibition of STAT/NF-κB signaling pathways (Zhang et al., 2015). Emodin – an anthraquinone derivative – significantly reduced ConA-induced nitric oxide (NO) production and the formation/release of Th1 (IL-2, IFN-γ, TNF-α) and Th17 (IL-6 and IL-17) cell cytokines—but induced those of Th2 (IL-4) and Treg (IL-10) cells (Sharma and Tiku, 2016). Taken together, the protective effect here of SHSST in EAE can be partially explained by the presence of the berberine, baicalin, baicalein, and emodin components of SHSST. However, the synergic effect among the other components may have been exerted to effect (although further studies are needed to elucidate this mechanism).

Corroborating these reports, the serum metabolites of SHSST and its three ingredients significantly decreased the ratios of IFN-γ to IL-4 in mitogen-stimulated mice spleen cells and human peripheral mononuclear blood cells (PBMCs) (Li et al., 2010). The results suggest that SHSST is a promising remedy for immunomodulation through Th1/Th2 regulation (Li et al., 2010). Nonetheless, the mechanism of SHSST activity with regards to inflammatory diseases remains unclear. In the present study, our findings demonstrated that pre-treatment and onset-treatment with SHSST delayed the onset or markedly alleviated the clinical severity of the EAE mice. Also, SHSST significantly decreased the population of CD4^+^ and CD4^+^/IFN-γ^+^ T cells and increased the population of CD4^+^/Foxp3^+^ T cells in the spinal cords from EAE mice. However, it did not significantly affect the population of CD8^+^, CD4^+^/IL-4^+^, CD4^+^/IL-17^+^ T cells. The results suggest that the protective effect of SHSST on the clinical severity of EAE in mice might be associated with the down-regulation of Th1 cells and the up-regulation of Treg cells, without its significant effects on Th2 and Th17 cells, in the spinal cords from EAE mice.

Demyelination in CNS induces permanent neurological deficits in patients with MS. The levels of demyelination and neurological deficits are closely connected with the degree of infiltration of inflammatory cells within or around lesions (Frohman et al., 2006; McFarland and Martin, 2007; Hoglund and Maghazachi, 2014). Thus, the use of herbal medicine to inhibit or regulate the activity of the inflammatory cells in the MS might be a new and exciting avenue of research. In the present study, onset-treatment with SHSST significantly reduced the level of demyelination and the infiltration of inflammatory cells including microglia, macrophage, and CD4^+^ T cells in the spinal cords from EAE mice. SHSST also reduced mRNA expression of representative chemokines (RANTES, MCP-1, and MIP-1α), cytokines (TNF-α and IL-1β), iNOS, and COX-2 – related with down-regulation of the NF-κB and MAPKs signaling pathways – in the spinal cords from EAE mice. Our results suggest that the protective effect of SHSST on demyelination might be associated with SHSST’s immunomodulatory and anti-inflammatory activities (although further studies are needed to confirm this hypothesis).

The peripheral autoreactive T cells against myelin antigen across the BBB and induce demyelination in CNS of patients with MS (Compston and Coles, 2008). Naive T cells are activated by contracting antigen-presenting cells (APC), such as dendritic cells, in secondary lymphoid organs and differentiate into Th cell subsets (Th1, Th2, Th17, or Treg cells) depending on different cytokine profiles and distinct effector function (El-behi et al., 2010; Chen et al., 2012). Th1 cells develop in response to IFN-γ and IL-12, express the defining transcription factor T-bet, produce the signature cytokine IFN-γ, IL-2, and TNF-α, which are involved in cell-mediated immunity against intracellular pathogens. Th2 cells develop in response to IL-4, IL-5, and IL-13; express GATA-3; secrete IL-4, IL-5, and IL-13; and mediate humoral immunity. Th17 cells differentiate in response to IL-1β, IL-6, IL-23, and TNF-α; secrete IL-17A, IL-17F, and IL-21; and play a critical pathogenic role in T cell-mediated autoimmune diseases (Reddy et al., 2004; Wraith et al., 2004; Chen et al., 2012). Treg cells are derived in response to TGF-β in the thymus; express Foxp3 (an important transcription factor of Treg cells), glucocorticoid-induced TNF receptor-related protein, cytotoxic T cell antigen 4, CD62L, and CD69; secrete TGF-β, IL-10, and IL-35; and play a central role in the maintenance of peripheral self-tolerance and immune homoeostasis. Therefore regulating differentiation, maturation, and activation of subtypes T cells is considered as a novel strategy for improving chemotherapy to treat autoimmune diseases, such as MS, although their exact roles are still poorly understood. In the present study, onset-treatment with SHSST significantly inhibited the increase in the population of CD4^+^/INF-γ^+^ T (Th1) cells in the spinal cords from EAE mice as well as the mRNA expression of IFN-γ in the spinal cord, while increased the population of CD4^+^/Foxp3^+^ T (Treg) cells and the mRNA or protein expression of Foxp3, TGF-β, and IL-10 in the spinal cord. However, SHSST did not significantly change the population of CD4^+^/IL-4^+^ (Th2) and CD4^+^/IL-17^+^ T (Th17) cells and the mRNA expression of IL-4, IL-5, IL-17, and IL-23 in the same organs. Our findings suggest that SHSST might alleviate the clinical symptoms of EAE through the down-regulation of Th1 cell response and the up-regulation of Treg cell response in the spinal cords from EAE mice, regardless of the alteration of Th2 and Th17 cell responses.

Treg cells suppress the differentiation and functions of Th1 cells *in vivo* and *in vitro*, which indicated that Treg cells have a profound therapeutic potential against diseases induced by Th1 *in vivo* (Xu et al., 2003; Taylor et al., 2006). However, the suppressive mechanism of Treg cells on Th1 cell differentiation remains unclear. In the recent study, Treg cells reduced the production of IFN-γ and the percentage of IFN-γ-, IL-2- and TNF-α-producing cells under Th1 cell culture conditions; and it suppressed the differentiation of T cells into Th1 cells (Shen et al., 2011). Additionally, the suppressive effects of Treg cells was recovered by the treatment of monoclonal antibody against TGF-β (an inducer of Treg cells) (Shen et al., 2011). The results suggest that the suppressive activity of Treg cells is mediated via TGF-β (Shen et al., 2011). Here, SHSST increased the mRNA or protein expression of Foxp3, TGF-β, and IL-10 and blocked the increase in the mRNA expression of IFN-γ, IL-2, and TNF-α in the spinal cords from EAE mice; whereas SHSST did not affect the mRNA expression of IL-4, IL-5, IL-17, and IL-23. In agreement with these results, bee venom acupuncture, electroacupuncture, dexamethasone, and acidic polysaccharide of Panax ginseng increased both the population of Treg cells and the expression of Foxp3 in the spinal cord or lymph organs; and were found to be associated with both the inhibition of Th1 cell responses and the delay of the onset and progression of EAE (Chen et al., 2006; Liu et al., 2010; Hwang et al., 2011; Lee et al., 2016c). Taken together, our findings strongly suggest that SHSST may exert a therapeutic role for MS by inhibiting Th1 cell’s response (but not Th2 and Th17 cell’s response), through the up-regulation of Treg cells’ response.

The immune response of MS is activated in the early effector phase, leading to the onset of disease. Activated auto-antigens in the onset phase induce secretion of chemokines/cytokines, including IFN-γ and TNF-α in the progressive phase of the disease. Therefore, quick treatment in the onset phase is very important for inhibiting the exacerbation of symptoms. Pre-treatment and onset-treatment of SHSST exerted a delaying or alleviating force with regards to the development and the progression of clinical signs and loss of body weight in EAE mice. The results suggest that SHSST has a preventive and therapeutic effect on early-stage EAE, although further studies are needed to understand the detailed mechanism by which SHSST operates, and thus how it might potentially prove useful as a therapeutic modality for the treatment of MS.

The BBB consist of pericytes, astrocytes, and a basal membrane. The BBB functions to protect the brain from toxins and microbes that may be present in the blood. It does this through the maintenance of tight, specialized intracellular junctions and the strict regulation of endocytosis (Fabis et al., 2007; Steiner et al., 2010; Pfeiffer et al., 2011; Kawakami et al., 2012). In MS and EAE, activated immune cells from the periphery, such as autoreactive T cells, may migrate into the CNS through the BBB and produce pro-inflammatory chemokines/cytokines leading to the activation of astrocytes, cellular adhesion molecules (ICAM-1, VCAM-1, etc.), and junctional molecules (zona occludens-1, claudin-3, claudin-5, etc.) between/or around the endothelial cells (Fabis et al., 2007; Steiner et al., 2010; Pfeiffer et al., 2011; Kawakami et al., 2012). In the present study, onset-treatment of SHSST inhibited the activation of astrocytes, reduced increased ICAM-1 and VCAM-1, and increased reduced zona occludens-1, claudin-3, and claudin-5 in the spinal cords from EAE mice. These results suggest that the onset-treatment of SHSST might contribute to the delayed or alleviated development and exacerbation of EAE by inhibiting the migration of immune cell into the CNS through preventing disruption of the BBB in EAE mice.

## Conclusion

Although an innovative medication to treat MS patients was unknown, a number of therapies – such as anti-inflammatory medications, interferon-beta, fingolimod, and alternative medicines – have been used to treat symptoms of MS (Mahdavian et al., 2010; Axisa and Hafler, 2016). Unfortunately, the therapies have either limited efficacy or adverse effects (Neilley et al., 1996; Gasperini and Ruggieri, 2009; Minagar, 2013; Finkelsztejn, 2014). Here, our findings are the first to suggest scientific evidence that SHSST can block both the development and the progression of EAE by inhibiting demyelination, microglial activation, macrophage infiltration, and expression of inflammatory mediators in spinal cords from EAE mice through/by the inhibition of the Th1 cell response and suppression of the Treg cell response. Additionally, SHSST has good bioavailability and can be given orally, which may reduce the costs of therapy (and the painful administration of an alternate drug), thus contributing to the patient’s adherence to the treatment regimen.

## Author Contributions

ML performed the behavioral experiment, immunohistochemistry, PCR analysis, flow cytometry analysis. ML and JC performed the Western blots analysis and prepared figures. SL contributed to study design and data interpretation. I-HC conceived all experiments, analyzed the results, and wrote the manuscript. All authors have read and approved the final manuscript.

## Conflict of Interest Statement

The authors declare that the research was conducted in the absence of any commercial or financial relationships that could be construed as a potential conflict of interest.

## Supplementary Material

DATA S1 **The effect of onset-treatment with SHSST on cytokines associated with Th17 and Th2 cells in the spinal cords from EAE mice. (A–D)** Lumbar spinal cord lysates were prepared from sham, EAE, EAE + SHSST, and SHSST groups at day 14–16 post-immunization. Each was analyzed for mRNA expression of IL-17 **(A)**, IL-23 **(B)**, IL-4 **(C)**, and IL-5 **(D)** by real-time PCR. Quantified data are expressed as mean fold induction ±SEM.
