# Supplementary figures and images for: Oriental Medicine Samhwangsasim-tang Alleviates Experimental Autoimmune Encephalomyelitis by Suppressing Th1 Cell Responses and Upregulating Treg Cell Responses
Source: Front Pharmacol. 2017 Apr 18;8:192. doi: 10.3389/fphar.2017.00192 (PMC5394181; doi:10.3389/fphar.2017.00192)

# Supplementary Data 1

■ Sham      ■ EAE      ■ EAE + SHSST      □ SHSST

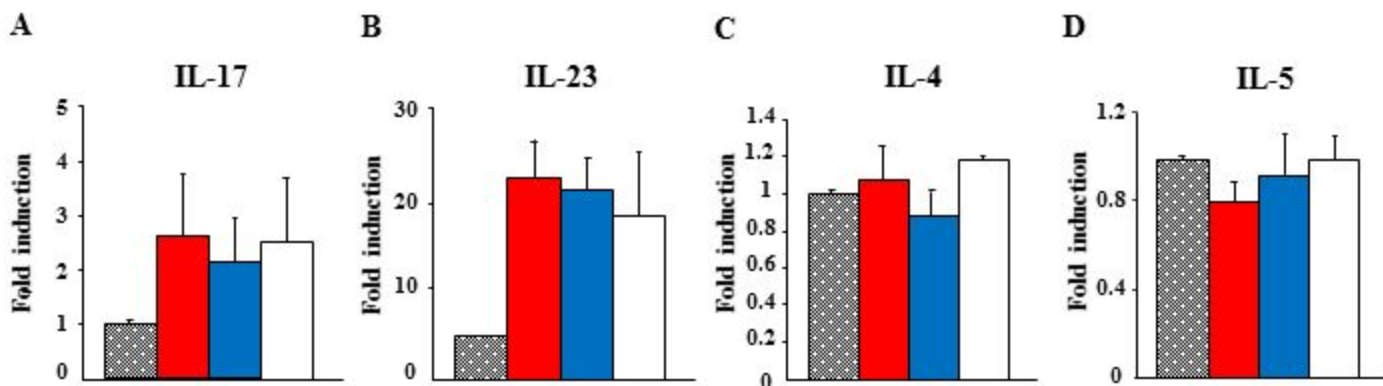

Supplement: DATA S1 — The effect of onset-treatment with SHSST on cytokines associated with Th17 and Th2 cells in the spinal cords from EAE mice. (A–D) Lumbar spinal cord lysates were prepared from sham, EAE, EAE + SHSST, and SHSST groups at day 14–16 post-immunization. Each was analyzed for mRNA expression of IL-17 (A), IL-23 (B), IL-4 (C), and IL-5 (D) by real-time PCR. Quantified data are expressed as mean fold induction ±SEM. [file Data_Sheet_1.PDF]
